# NCI's provocative questions on cancer: some answers to ignite discussion

**DOI:** 10.18632/oncotarget.432

**Published:** 2011-12-31

**Authors:** Mikhail V. Blagosklonny

**Affiliations:** ^1^ Department of Cell Stress Biology, Roswell Park Cancer Institute, BLSC, L3-312, Elm and Carlton Streets, Buffalo, NY, 14263, USA

**Keywords:** NCI, cancer, therapy, prevention, aging, rapamycin, mTOR

## Abstract

National Cancer Institute has announced 24 provocative questions on cancer. Here I try to answer some of them by linking the dots of existing knowledge.

## INTRODUCTION

As announced by the NCI director Harold E. Varmus, answers to these perplexing questions will revolutionize prevention and treatment of cancer. How does obesity contribute to cancer risk? What is the mechanism by which some drugs commonly and chronically used for other indications protect against cancer? Can we use our knowledge of aging to enhance prevention or treatment of cancer? Can we develop methods to rapidly test interventions for cancer treatment or prevention? Why are some disseminated cancers cured by chemotherapy alone? Can we extend patient survival by using approaches that keep tumors static? Why do many cancer cells die when suddenly deprived of a protein encoded by an oncogene? http://provocativequestions.nci.nih.gov/?cid=WTq_cgov

What these questions have in common is that they cannot be answered by a single experiment. Knowledge from different fields needs to be brought together and seemingly unrelated facts to be linked. Then predictions can be tested by retrieving published data (virtual experiments) [1, 2]. Here are answers to some questions. Since the order of questions was arbitrary, I have re- arranged questions, keeping the original numbers.

### PQ-22: Why do many cancer cells die when suddenly deprived of a protein encoded by an oncogene?

Oncogene addiction is dependence on oncogene, even though this oncogene was not needed before its activation [3-31]. For example, transfection of Bcr-Abl renders HL-60 cells apoptosis-reluctant, resistant to killing by most anti-cancer drugs [28, 32, 33]. In contrast, the Bcr-Abl inhibitor imatinib kills Bcr-Abl-transfected cells without affecting parental cells. Parental cells neither have Bcr-Abl nor need Bcr-Abl to start with. So why losing Bcr-Abl is detrimental but not having Bcr-Abl at all is not. Bcr-Abl inhibits apoptosis and therefore some other anti-apoptotic proteins become redundant. For example, while Bcl-2 is over expressed in HL-60 cells, it is not expressed in HL60/Bcr-Abl cells [34, 35]. (By the way, this also explains why Bcl-2 (and p53) status does not correlate with cell propensity to apoptosis (see [36-38]).

The Bcr-Abl addiction can be described by the dam model [39]. Bcr-Abl is ‘a dam on the pro-apoptotic river’. Pro-apoptotic molecules accumulate upstream of the dam. For example, hyper-active caspase-9 was detected in Bcr-Abl-expressing HL-60 cells [40]. When Bcr-Abl is suddenly removed, then apoptotic signals “flow” downstream, causing a flood [39, 40].

**Figure 1:**
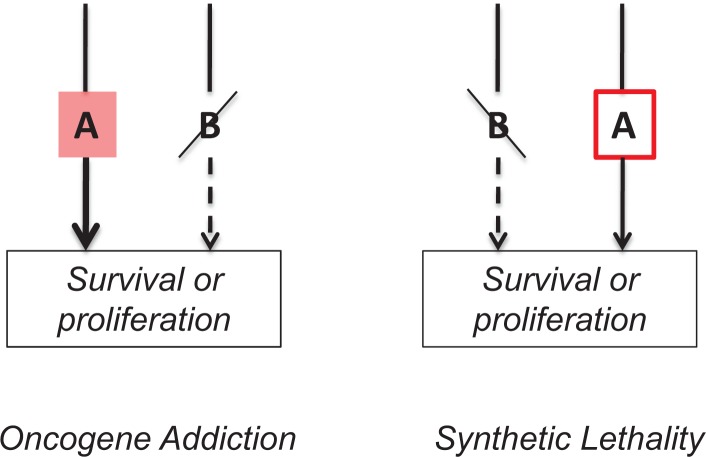
Oncogene addiction and synthetic lethality Oncogene addiction Activation of pro-survival pathway A leads to deactivation of parallel (and redundant) pro-survival pathway B. Cell becomes addicted to “A”. Targeting “A” will kill this cell. **Synthetic lethality.** Loss of pro-survival pathway B renders the cell dependent on pro-survival pathway A. Targeting “A” will kill this cell.

Let us make a generalization: Activation or over-activation of a pro-survival pathway may lead to deactivation of an alternative (and redundant) pro-survival pathway(s) because of redundancy (Figure 1, oncogene addiction).

Now we can connect two dots: Oncogene addiction (OA) and synthetic lethality (SL). Two genes are synthetic lethal if mutation of either alone is compatible with viability but mutation of both leads to death [19, 41-44]. At first glance, OA and SL are different phenomena. Yet, the difference between OA and SL is the sequence of events and … our knowledge about these events.

In synthetic lethality, gene B (or process B) is inactivated first (Figure 1). This renders cell dependent on gene A (or process A). In oncogene addiction, gene A is overactivated first and gene B is inactivated later. Oncogene addiction (OA) is a mirror image of synthetic lethality (SL). The distinction between SL and OA depends on our knowledge of the sequence of events. When we introduce an oncogene, this is oncogene addiction. But what about natural oncogene-dependent tumors. Is that OA or SL? We cannot distinguish them. In other words, OA is SL and vice versa, depending on our point of view. For example, in OA gene A is known. In SL, we screen for gene A using agents that toxic to such cells. (Note: instead of gene, there might be a pathway or a process such as glycolysis, oxidative or protetoxic stress [45-47]. We use the word gene for brevity.)

In natural tumors, oncogene addiction is a consequence of selection for resistance to restrictive, growth-limiting conditions, when resistance is conferred by oncogene A. Definition: oncogenic resistance is resistance to cytostatic/cytotoxic agents based on oncogenic alterations such as loss of p53 or expression of Bcr-Abl, which renders cells both resistant and malignant [48]). But then the oncogenic cell may lose redundant pathway/gene B, therefore becoming addicted to oncogene A. Thus, oncogene addiction (or synthetic lethality) and oncogenic resistance are two sides of the same coin.

### PQ-21: Given the appearance of resistance in response to cell killing therapies, can we extend survival by using approaches that keep tumors static?

Will static drugs cause resistance? The answer is yes. Anything that is cytostatic must select for resistance. Sensitive cells get arrested, whereas rare resistant cells selectively proliferate. But this does not preclude successful therapy. Furthermore, selection for resistance is a consequence of successful treatment that keeps most cells static. In comparison, antibiotics (especially cytostatic agents) effectively select for resistance in bacteria. But still antibiotics are very useful. Compared with bacteria, mutation rates and cell numbers and mutation rate are relatively lower in cancer and resistant cancer cells do not spread to new patients (so acquired resistance always is acquired de novo).

While arresting sensitive cancer cells, static drugs select for resistant cancer cells. So therapy that effectively arrest tumor growth must select for resistance. Fortunately, any anticancer drugs including static drugs can select for resistance, only by causing therapeutic response due to arresting or killing non-resistant cells. Unfortunately, when resistance arises, resistant cancer cells tend to be more malignant and aggressive. This is because resistance can be oncogenic [48]. Therefore, therapeutic response often does not prolong life of cancer patient [49].

But here is a solution. In resistant tumors, we can select for drug sensitivity using antagonistic drug combinations [50]. Antagonistic combinations may be comprised of a toxic drug and a static drug that antagonizes (blocks) the toxic drug. If cells are (or become) resistant to the static drug, the toxic drug will kill them. Of course, the entire combination selects for resistance but this is resistance to the entire combination [50]. Yet resistance to the entire combination can be achieved by selection for sensitivity to the static drug. Such antagonistic combinations selectively eliminate resistant cancer cells in cell culture [51-56].

Furthermore, if normal cells are sensitive to a static drug but resistant cancer cells are not, then resistant cancer cells could be eliminated without toxic side effects. This links two dots: (a) prevention of resistance by antagonistic combinations and (b) protection of normal cells from chemotherapy. Normal cells can be protected from cell cycle-dependent chemotherapy by pre-treatment with cytostatic agents [56-62] and also by agents that block cell death selectively in normal cells [54, 63-68]. This strategy was discussed in detail [69-72]. Most importantly, drug combinations that selectively kill resistant cancer cells, while sparing normal cells, can be designed using currently available drugs [62, 71, 73].

So will static drugs cause resistance? The answer is yes. But can they extend patient survival (despite appearance of resistance). The answer is also yes. Yes, if additional therapeutic modalities (such as antagonistic combinations and protection of normal cells) will be used in sequence [74].

### PQ-19: Why are some disseminated cancers cured by chemotherapy alone?

Let us first discuss why most disseminated cancers are NOT cured by chemotherapy alone? First, common cancers such as lung, colorectal, breast, prostate, pancreatic, renal, thyroid cancers are age-related diseases, which occur late in life. During lifespan pre-malignant and malignant cells acquire mutations, undergo multiple rounds of selection and replication [75-80]. Cancer cells accumulate hundreds of mutations that render them oncogenic, abolishing cell death and cycle arrest [78, 81-92]. Not surprisingly, they are also intrinsically resistant to chemotherapy. Furthermore, chemotherapy itself causes selection for resistance, which is associated with more malignant and aggressive phenotype and acceleration of tumor growth [49, 93, 94].

Second, these cancers arise from normal tissues resistant to therapy to start with. For example, side effects of chemotherapy are not prominent in breast and lung tissues compared with bone marrow. Why would breast and lung cancer be more sensitive to chemotherapy than bone marrow? Even further, apoptosis-avoidance is a hallmark of cancer, so these cancer cells must be even more resistant than their normal counterparts. Not surprisingly, therapeutic window is low. (Note: as we discussed, oncogenes confer resistance to some chemotherapy on the cost of oncogene addiction, which could be exploited for therapy).

Third, metastasis may require mutations beyond those required for primary tumors [82]. These additional oncogenic changes may contribute to resistance of disseminated cancers. For example, in pancreatic cancer, at least a decade separates the occurrence of the initiating mutation and the birth of the parental, non-metastatic founder cell. And at least five more years are required for the acquisition of metastatic ability and patients die an average of two years thereafter [95]. So cancer cells undergo long selection for fitness and oncogenic resistance [80, 96].

However, some cancers are highly sensitive to chemotherapy. Examples include testicular cancer, gestational choriocarcinoma, some lymphomas, childhood malignancies such as Wilms tumors. These cancers share three features.

1. Curable cancers arise from apoptosis-prone tissues such as lymphoid, testicular, embryonic and placental/endometrial.

Apoptosis is a marker of therapeutic response and curable malignancies are prone to undergo apoptosis in response to therapy [97-105]. (Note: it has been emphasized that in most cancers apoptosis is not a predictive marker of therapeutic response [106-109]. Apoptosis is not important for therapy of such cancers, simply because these cancers are apoptosis-reluctant [110, 111]. These are the same common cancers that are not curable by chemotherapy alone. One may suggest that these cancers are not curable exactly because apoptosis is not a primary response to chemotherapy or in other words because they are apoptosis-reluctant.

In contrast, curable disseminated cancers are apoptosis-prone. Testicular germ cell tumors are unique in their excellent response to DNA-damaging chemotherapy. Hypersensitivity of testicular tumors to etoposide-induced apoptosis is associated with functional p53 [112]. In cancer with overexpressed Mdm2, nutlin-3a induces p53 and apoptosis [113]. Similarly, testicular cancer easily undergo apoptosis in response to p53 induced by cisplatin. Resistance to cisplatin is linked to p53 mutation [114, 115]. Relapsed tumors are resistant to therapy [116].

2. Curable cancers arise without lengthy selection and progression. A few mutations may be sufficient for dissemination of these particular cancers (see feature 3) but they did not acquire resistance associated with tumor progression.

A peculiar example of cancer-like condition represents endometriosis, growth of normal endometrial cells, resembling malignant processes, including invasive growth and distant implantation. Oncogenic mutations are absent or very rare [117]. Medulloblastoma, the most common malignant brain tumor of children [118] has lower genetic alterations compared to adult solid tumors.

3. Curable disseminated cancers arise from tissues that “normally metastasize” (hematopoietic/lymphoid) and invasive (the placenta) and highly proliferative. So they can become disseminated with minimal number of mutations and without tumor-progression.

### PQ-17: Since current methods to assess potential cancer treatments are cumbersome, expensive, and often inaccurate, can we develop other methods to rapidly test interventions for cancer treatment or prevention?

Although different cell culture methods can be suggested, they probably would share something in common: these rapid methods will be unaesthetic.

Currently, many methods are based on evenly plated cells in relatively low cell densities. In control, cells are beautifully healthy. It is easy to observe spectacular effects of drugs that induce apoptosis and senescence. However, almost everything in these methods is artificial. (the only correct parameters are temperature and CO2 levels.)

a. In the organism, cells exist in very high densities.

b. 21% oxygen used in cell culture does not exist in the body, only in the air. In tumors oxygen levels are 0. 1-3%.

c. Cancer cells are usually cultured in high-glucose DME, with levels of glucose 5 fold higher than blood glucose levels.

d. In real tumors, levels of lactate are very high and pH is low.

Yet, hypoglycemic/hypoxic condition in vitro mimicking the tumor microenvironment markedly reduced the efficacy of anticancer drugs [119]. In the high cell density in hypoxia model, cancer cells lose viability due to self-poisoning with lactic acid. Some anti-cancer agents actually increase cell viability [120]. Overgrown, “yellow” cell cultures in hypoxic conditions may mimic in vivo environment.

### PQ-7: How does the lifespan of an organism affect the molecular mechanisms of cancer development, and can we use our deepening knowledge of aging to enhance prevention or treatment of cancer?

What are cellular and molecular mechanisms linking aging and cancer? And what is cellular aging?

In proliferating normal cells, growth factors (GF) stimulate (a) cellular mass growth and metabolism and (b) cell cycle progression. Cellular mass growth is balanced by cell division. (Many signaling pathways that promote mass growth and metabolism converge on mTOR, so I will refer to them as the mTOR network or pathway). In the absence of growth signals, the cell neither grows nor cycles. This is quiescence [121, 122]. When the cell cycle is blocked but mTOR is still active, then the arrested cell becomes senescent [122-134]. mTOR renders cells resistant to insulin and growth factors. As discussed in detail, senescent cells are hypertrophic, hyperfunctional, overactivated, pro-inflammatory and hypersecretory, signal-resistant and lack the regenerative potential (the inability to restart proliferation). Thus, mTOR converts quiescence into senescence. This process could be called gerogenic conversion or geroconversion [122]. Rapamycin slows down geroconversion. Rapamycin is a gerosuppressant.

Furthermore, mTOR is involved in cell senescence and stem cell exhaustion in the organism [135-140]. Also, rapamycin reverses cellular phenotypes in Hutchinson-Gilford progeria syndrome cells [141]. Whereas calorie restriction (CR) deactivates the nutrient-sensing mTOR pathway [142], short-term CR suppresses cellular senescence in the organism [143, 144].

There are 3 links between aging and cancer that are in part mTOR-dependent.

First, senescent cells secrete pro-inflammatory factors [145-154]. Second, mTOR overactivation can cause insulin resistance [155-159], which in turn leads to a compensatory increase in insulin levels, which can promote cancer. Third, signal-resistance, irresponsiveness and loss of regenerative potential of the aging normal cells create a selective pressure to bypass the need for growth-signals and bypass cell cycle block. Unable to respond to physiological stimuli, normal cells are in disadvantage, unable to compete with premalignant cells. Cells with oncogenic mutations and loss of cell cycle control (due to mutations in p53, p16 and Rb) selectively proliferate. In other words, due to irresponsiveness of aging normal cells to mitogenic signals and decreased regenerative potential of aging cells, there is a selective advantage for transformed cells, which are autonomous and lack cell cycle checkpoints. For example, declining lymphoid progenitor fitness promotes aging-associated leukemogenesis [160, 161]. I suggest that restoration of signal-sensitivity and responsiveness of normal cells by pulse (intermittent) treatment with rapamycin can abolish selective advantage for cancer cells.

Thus, at least 3 mechanisms of how mTOR-driven aging can contribute to cancer. This predicts that suppression of aging of normal cells by rapamycin will extend lifespan and delay cancer. In fact, numerous data support this prediction (see PQ-5). Noteworthy, rapamycin is not intended to directly affect cancer cells. It is intended to suppress geroconversion (suppress aging of normal cells). As a gerosuppressant, rapamycin will be used in low doses and in pulses, thus precluding side effects [162, 163].

In summary, the incidence of common cancers such as breast, prostate, colon, lung, pancreatic, gastric, bladder and certain leukemias is increased with age. Conditions that accelerate aging such as obesity also accelerate cancer, whereas slow aging is associated with delayed cancer. One can suggest that pharmacological interventions that slow down organismal aging will delay or prevent cancer. It was demonstrated that mTOR is involved in cellular senescence, converting quiescence into senescence (geroconversion). Importantly, mTOR is involved in organismal aging and its inhibition extends lifespan. Aging can be decelerated by rapamycin.

### PQ-5: Given the evidence that some drugs commonly and chronically used for other indications, such as an anti-inflammatory drug, can protect against cancer incidence and mortality, can we determine the mechanism by which any of these drugs work?

Some drugs commonly and chronically used for other indications can protect against cancer. As announced by NCI, elucidating the mechanisms by which these agents work would be a major breakthrough in cancer prevention.

Preclinical and clinical data suggest that certain drugs used for diabetes, hypertension, atherosclerosis, inflammation and immunossupression can protect against cancer. These drugs include metformin, beta-blockers, angiotensin-blockers, aspirin and rapamycin. Since type II diabetes, hypertension, pro-inflammation and atherosclerosis are all age-related diseases and conditions, we can expect that these drugs may affect the aging process. And since cancer is also an age-related disease, conditions that slow down aging in turn delay or prevent cancer. At doses used in the clinic for treatment of age-related diseases, these accidental cancer-preventive agents are relatively ineffective to treat cancer, implying that their cancer-preventive effects are not due to targeting cancer cells directly. Since the mTOR pathway is involved in cellular and organismal aging and age-related diseases, one can suggest that cancer preventive activities of “accidental” drugs are in part due to suppression of aging.

**Rapamycin** decelerates geroconversion (conversion of quiescence into senescence) in arrested cells [122-132]. Also rapamycin suppresses yeast aging and prolongs life span in Drosophila and mice [164-183].

Finally, rapamycin prevents cancer in mice [178, 179, 184-192] and humans [193-196]. Given that mTOR is the only one target of rapamycin, one can conclude that inhibition of mTOR is sufficient to suppress aging and delay cancer.

**Metformin**, an anti-diabetic drug, inhibits the mTOR pathway [197-200]. Metformin and its analog phenformin slow down aging, delay cancer and extend life span in rodents [201-210]. Also metformin decreases the risk of cancer in humans [206, 211-220].

**Angiotensin-II-blockers**. Inhibitors of angiotensin II activity include ACE inhibitors (such as captopril and lisinopril), which decrease angiotensin II production, and angiotensin receptor blockers such as losratan. Angiotensin-II-blockers suppress chemically-induced colon carcinogenesis in obese mice [221], hepatocarcinogenesis in rats [222] and metastasis in mice [222-224]. In humans, use of these drugs is associated with a lower incidence of cancer occurrence [225, 226]. In patients with renal transplantation, the use of angiotensin-II-blockers is associated with a two-fold reduced risk of skin cancers [227].

Angiotensin-II activates mTOR pathway and causes cellular hypertrophy [228-236]. Therefore, angiotensin-II-blockers, which prevent these effects, are indirect inhibitors of mTOR.

**Beta-blockers**, which are used for therapy of hypertension, prevent breast cancer [237-242]. There are several publications that activators of beta-androgenic receptors can activate the mTOR pathway [243-245]. Therefore, beta-blockers are expected too block mTOR activation. This requires further investigations.

**Aspirin** decreases cancer incidence in humans [246-252]. As an anti-inflammatory agent, it decreases an important hallmark of aging. The effect of aspirin on gerogenic-signaling pathways such as mTOR needs to be studied in the organism. In some cell models, salicylate inhibits phosphorylation of S6, a downstream target of mTOR/S6K [253].

### PQ-1: How does obesity contribute to cancer risk?

Summary: The simplest answer is that obesity promotes cancer by over-activating the nutrient-sensing mTOR pathway, which is involved in obesity, aging and cancer. Cancer is an age-related disease and accelerated aging promotes cancer. High-calorie diet and obesity activate mTOR, thus promoting aging and cancer. Rapamycin increases lifespan in mice including cancer-prone mice and prevents cancer in part by slowing down aging. Given that rapamycin is a clinically approved drug, it can be used in low doses to prevent cancer in obese patients. Thus, one can suggest not only how obesity contributes to cancer risk but also a therapeutic strategy for cancer prevention.

*How obesity and cancer might be linked*

Many studies have documented an increased risk of cancer incidence and mortality in individuals who are obese [254-260]. What are mechanisms that underlie this risk? There are causative and correlative links between obesity and cancer.

Causative links: obesity promotes cancer

First, several factors secreted by the adipose tissue can directly stimulate tumor growth. Second, obesity causes hormonal changes such as insulinemia and insulin promotes cancer. Third, as we will discuss, obesity can accelerate aging and aging promotes cancer.

Correlative links: both obesity and cancer are promoted by a common cause

**Figure 2:**
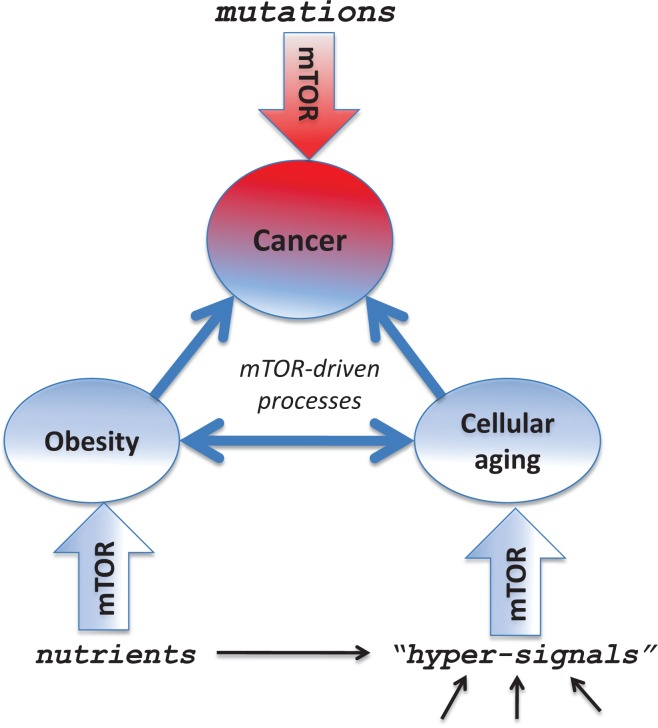
Several mTOR-dependent processes acting in concert can promote cancer The mTOR pathway is involved in cellular and organismal aging, thus connecting aging to age-related diseases such as cancer. Pro-aging, growth-promoting and inflammatory pathways such as mTOR drive aging and cancer. Rapamycin may decrease cancer by (a) slowing aging, (b) preventing obesity and (c) directly affecting cancer cells.

First, aging is a major risk factor for cancer and is associated with visceral obesity. Second, high-calorie diet can promote both obesity and cancer. Yet, even these correlative relationships are causative on a deeper level, sharing a common molecular mechanism that links aging, cancer and obesity. Thus, the same pathway (such as mTOR) may be involved in aging, obesity and cancer per se, as well as aging and obesity can mutually stimulate each other (via the mTOR pathway) and both of them play causative role in cancer (Figure 2). Pharmacological inhibition of such a common pathway will prevent or delay cancer. What are molecular changes induced by obesity that actually promote cancer development?

**Dot 1**. Nutrients and insulin activate mTOR, whereas calorie restriction (fasting) deactivates mTOR [142, 156, 261-266].

**Dot 2**. The mTOR pathway promotes obesity and is activated in obesity [256, 261, 265, 268-272].

**Dot 3**. mTOR is involved in cellular aging [122-132, 136-139, 273] and organismal aging [164-183]. Noteworthy, basal (fasting) levels of mTOR activity is increased in old mice [266].

Taken together (dots 1-3) these data predict that obesity would accelerate aging and age-related diseases, thus shortening life span. This prediction does not need to be tested. It is too well known that: Obesity accelerates all age-related diseases.

**Dot 4**. Obesity accelerates age-related diseases and shortens lifespan.

**Dot 5**. Cancer is age-related disease and accelerated aging accelerates cancer. Prediction 2. Since obesity accelerates aging and age-related diseases, obesity must accelerate cancer, which is an age-related disease. This prediction does not need to be tested. It is too well known that: Obesity increases cancer risk. Furthermore, this is exactly the starting point (PQ-1).

Prediction 3: Rapamycin should delay cancer by slowing down the aging process. In fact, rapamycin prevents cancer in mice [178, 184-192] and humans [193-196].

Noteworthy, the activation PI3K/mTOR pathway by mutations (Figure 2) is one of the most universal alterations in cancer [87, 274-284].

As a gerosuppressant, rapamycin will be probably used in low doses and intermittent schedules to avoid side effects. It could be used for cancer prevention in obese patients with multiple age-related pathologies and could be combined with diet, physical exercise, aspirin, metformin, beta-blockers, angiotensin-blockers and lipid-lowering drugs. Then cancer can be delayed by staying young.
